# Epigenetic Guardian: A Review of the DNA Methyltransferase DNMT3A in Acute Myeloid Leukaemia and Clonal Haematopoiesis

**DOI:** 10.1155/2017/5473197

**Published:** 2017-02-14

**Authors:** Sabah F. Chaudry, Timothy J. T. Chevassut

**Affiliations:** Brighton and Sussex Medical School, University of Sussex, Brighton, East Sussex BN1 9PS, UK

## Abstract

Acute myeloid leukaemia (AML) is a haematological malignancy characterized by clonal stem cell proliferation and aberrant block in differentiation. Dysfunction of epigenetic modifiers contributes significantly to the pathogenesis of AML. One frequently mutated gene involved in epigenetic modification is DNMT3A (DNA methyltransferase-3-alpha), a DNA methyltransferase that alters gene expression by de novo methylation of cytosine bases at CpG dinucleotides. Approximately 22% of AML and 36% of cytogenetically normal AML cases carry DNMT3A mutations and around 60% of these mutations affect the R882 codon. These mutations have been associated with poor prognosis and adverse survival outcomes for AML patients. Advances in whole-exome sequencing techniques have recently identified a large number of DNMT3A mutations present in clonal cells in normal elderly individuals with no features of haematological malignancy. Categorically distinct from other preleukaemic conditions, this disorder has been termed clonal haematopoiesis of indeterminate potential (CHIP). Further insight into the mutational landscape of CHIP may illustrate the consequence of particular mutations found in DNMT3A and identify specific “founder” mutations responsible for clonal expansion that may contribute to leukaemogenesis. This review will focus on current research and understanding of DNMT3A mutations in both AML and CHIP.

## 1. DNA Methylation

### 1.1. Epigenetic Modifications

Normal haematopoiesis is one of many cellular processes regulated by epigenetic modification. Haematopoietic stem cells (HSCs) are required to proliferate, differentiate, and mature in a controlled fashion down various distinct myeloid and lymphoid lineages giving rise to terminally differentiated blood cells. Due to the varied number of lineages that can arise from haematopoietic stem cells, dysfunction produces an assortment of disease phenotypes. Aberrant expressions of epigenetic regulators are increasingly recognised as being central to this process.

Two major forms of epigenetic regulation are recognised. DNA methylation involves direct modification of the DNA molecule itself via the addition of a methyl group to cytosine bases, generally at CpG dinucleotides, without any actual alteration of the DNA sequence itself, changing how the DNA is read and influencing the level of expression of the gene. A second level of epigenetic regulation occurs at the level of the histones which can undergo various chemical modifications including methylation, phosphorylation, and acetylation, thereby influencing local transcriptional activity. In this report, we discuss DNA methylation and its role in haematopoiesis and haematological malignancy with particular focus on DNMT3A.

### 1.2. DNA Methylation

**Figure 1:**
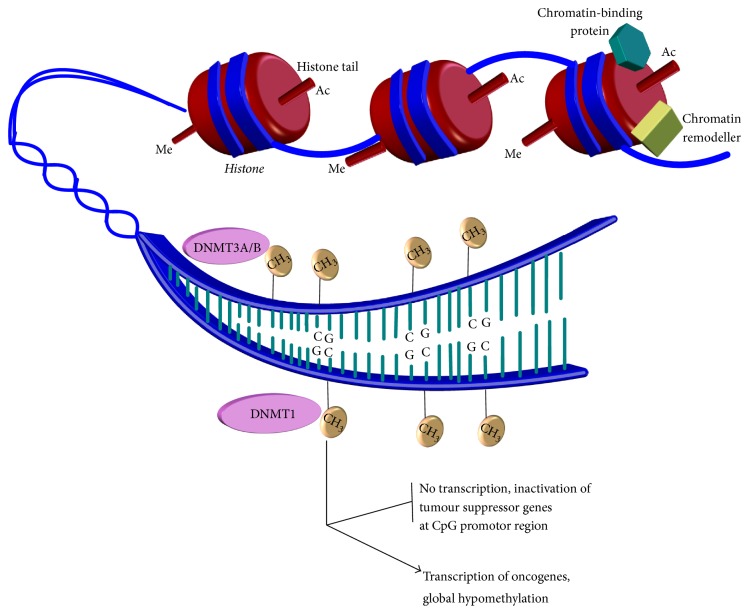
DNA methylation by DNA methyltransferase enzymes. An illustration showing the positively charged histones binding the negatively charged DNA into compact chromatin to prevent gene transcription. The figure shows how other proteins can interact with histones to regulate transcription of genes. Modifications of histones tails such as acetylation and methylation change chromatin architecture, unwinding chromatin to allow access to the DNA sequence. Several other proteins, including chromatin remodellers, can also affect chromatin architecture. Regulators such as DNA methyltransferase enzymes are then able to access DNA to add methyl groups (CH_3_) to appropriate cytosine bases. The methyl group is added to the C5 position of the pyrimidine ring to produce 5-methylcytosine (5mC). Aberrant methylation as illustrated can inactivate tumor suppressor genes (through hypermethylation) and increase expression of oncogenes (through hypomethylation of promotor sites of these genes), both of which can contribute to leukaemogenesis.

DNA methylation is an important process involved in developmental patterning, chromatin modification, and imprinting. Aberrant methylation has long been recognised to have a role in disease processes such as cancer [1, 2]. As Figure 1 shows, methylation involves the addition of a methyl group (CH_3_) to specific cytosines base found in the DNA sequence to produce 5-methylcytosine (5mC) [3].

Methylation occurs at sites in the genome where the cytosine base is bound to the adjacent guanine base by a phosphodiester bond. These sites are referred to as CpG residues and human genes have approximately 60–80% of these individual CpGs methylated [4]. However, clustered CpG residues in “islands” are often located near gene promoter regions and are predominantly nonmethylated [4]. This allows for important regulation of gene expression. While highly studied, the purpose and function of these DNA methylated promotor sites are not well understood. Previous studies have highlighted that the density of CpG residues have been consequently related to methylation levels. Promoter sites that are poorly populated with CpG residues are seen to be highly methylated, while the opposite has been observed in promotor sites with highly populated CpG residues [5]. While studies have suggested a correlation between promoter methylation and transcriptional activity, with methylated promoters often being transcriptionally silent [4, 6–9], it is not clear that DNA methylation itself is responsible for gene silencing, and rather regulation of gene expression through DNA methylation may be far more complex.

New methods in genome-wide mapping have allowed for further insight into the role of gene regulation by DNA methylation. A growing body of evidence suggests that, rather than working in isolation, DNA methylation could possibly be intertwined with other gene silencing mechanisms. Work specifically looking at the modulation of chromatin has produced various theories on the functional role of CpG island methylation at promotor sites in respect to gene expression. One hypothesis surrounding the role of DNA methylation is that it may modulate polycomb protein targeting [10]. Several studies gave early indications that PRC2 (polycomb repressor complex 2) played a key role by observing that PRC2 components were binding to CpG islands [11–13]. Specifically the histone marker H3K27me3, a trimethylated histone, has been used in studies as a signature of PRC2 activity and has also been identified to cause gene silencing through several mechanisms [14]. Interestingly, a negative correlation has been suggested to exist between DNA methylation, H3K27me3, and CpG high density regions [15, 16]. This has led to the theory that DNA methylation may negatively regulate the targeting of polycomb proteins [10]. Data to support this is still correlative, where global levels of hypomethylation are associated with increased PRC2 binding [11, 14, 15], but further work is needed to establish the temporal and spatial relationship between DNA methylation and polycomb targeting and regulation of gene expression.

Though the canonical regulation of promotor sites by methylation is an important phenomenon observed in X-chromosome inactivation and imprinting [1, 17], the actual role of methylation in developmental processes and embryonic patterning is not well understood. While hypomethylated promotor sites are found to be present at important germline specific genes during development, these genes are also found to be methylated in somatic cells [5]. Though studies have shown that the absence of methylation in mouse embryo models have led to the activation of specific genes [18, 19], it is unclear whether the methylation process itself is directly involved in gene silencing in embryonic patterning and highlights the complex role of methylation in developmental processes. Methylation of nonpromoter regions appears to be largely irreversible and this epigenetic memory and irreversibility are an important property of gene silencing in early development to produce monoallelic expression of specific genes [20]. In contrast to this, plasticity of methylation patterns at promotor sites has been suggested to regulate cellular properties such as pluripotency in HSCs to prevent uncontrolled differentiation [18, 20, 21].

The identification of areas flanking CpG islands, called “shores” and “shelves,” adds further complexity to the regulation of gene expression. Shores are identified as being 0–2 kb away from islands, while shelves are 2–4 kb away; furthermore, “open sea” sites, which are spread throughout the genome itself, have also been identified [22, 23]. Aberrant levels of hypomethylation and hypermethylation of these shores, shelves, and open sea sites have been associated with several malignant processes [2]. However, the significance of such areas and the pattern of methylation observed in these disease processes at these sites is still unclear. Recent studies investigating HSCs have identified hypomethylated regions referred to as “canyons” [24]. These canyons may overlap CpG islands but are characteristically distinct from low-methylated islands [24]. Canyons are seen to be conserved across cell types and species. Genes associated with haematopoiesis are reported to be enriched in canyons and are vulnerable to methylation dysfunction observed in haematological malignancy [24].

Global hypomethylation and associated overall genomic instability in DNA elements such as exons, introns, and transposons have been suggested to have a role in disease processes [9, 25]. Altered levels of methylation have been associated with instability of microsatellite repeats; dysfunction of such repeat elements is well characterized in diseases such as Huntington's disease and cancer [9]. In addition to hypomethylated promotor sites being transcriptionally active, global hypomethylation has also been associated with the activation of new enhancer sites, similarly resulting in gene upregulation [26].

### 1.3. DNA Methyltransferase (DNMT) Enzymes

**Figure 2:**
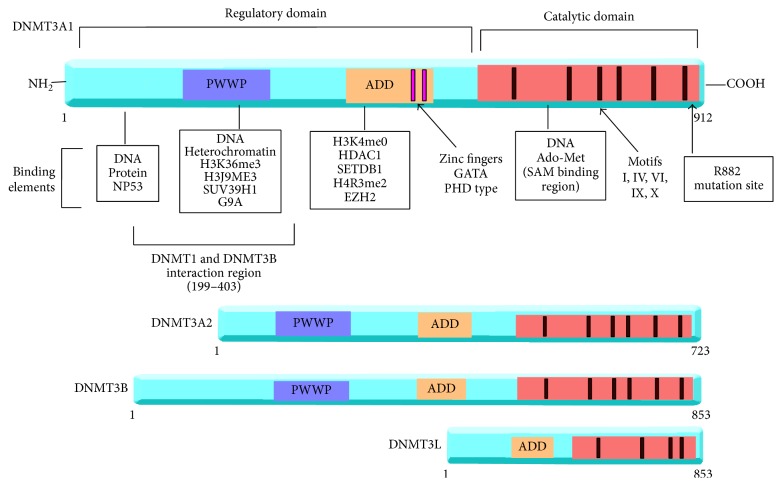
Structure of DNMT3A splice isoforms, DNMT3B, and DNMT3L. Shown here is the structure of the DNMT3 enzymes. The ADD domain is related to the PHD- (plant homeodomain-) like regulator ATRX and has strong interactions with histones, which is thought to enhance its methylation activity. Meanwhile PWWP domain (Pro-Trp-Trp-Pro) is found to interact with DNA and heterochromatin to help carry out its function, among other proteins. The catalytic domain of the enzyme has motifs conserved across the isoforms. Motifs I are cofactor binding while motifs VIII and IX are for DNA binding and methylation activity at motifs IV, VI, and VIII. The main difference between the two splice isoforms of DNMT3A1 and DNMT3A2 is the extra DNA binding domain located at the amino terminal of DNMT3A1. Other DNMT enzymes are also able to interact with DNMT3A. One common mutation site shown here is R882 residue. This is a hotspot for mutations in haematological malignancy and preleukaemic conditions. Not depicted here are the splice isoforms for DNMT3B and the structure of DNMT1. Adapted from Yang et al. [2].

DNA methylation is carried out by a family of DNA methyltransferase enzymes, which include DNMT1, DNMT3A, and DNMT3B.  Figure 2 illustrates the structure of these genes. By contrast, DNMT3L has no catalytic domain and predominantly functions as an accessory protein to DNMT3A [29]. Studies have suggested that DNMT3L may work to localise DNMT3A to chromatin sites in developmental processes and is also seen to interact with DNMT3B in vivo [29].

The methylation of existing genomic CpG islands is carried out by DNMT1 (see Figure 1). Several studies have identified the importance of this enzyme. Dysfunction of DNMT1 has been shown to contribute to various different types of malignancies such as colorectal and prostate cancer [30]. In knockdown mice models, DNMT1^(−/−)^ demonstrated a 90% loss of methylation and was associated with embryonic lethality suggesting its incompatibility with life [30, 31]. Though viable, DNMT1^(−/−)^ embryonic mice stem cells resulted in loss of important cellular function such as differentiation [32]. The loss of DNMT1 and subsequent loss of genomic stability and viability of cellular function [31] are all characteristics noted in many malignant processes.

The methylation of de novo (new) genomic sequence is carried out by both DNMT3A and DNMT3B (see Figure 1) [29, 33]. The knockdown of both DNMT3b^(−/−)^ and DNMT3a^(−/−)^ mice embryonic stem cells are not observed to be compatible with life due to the extensive disruption of normal developmental process [33], confirming a crucial role of DNMT3 enzymes in embryonic development.

### 1.4. TET Enzymes

While the addition of methyl group is carried out by DNMT enzymes, the removal of the methyl group and conversion to unmodified state occur through both the inhibition of DNMT enzymes and also by the action of a group of enzymes known as Ten-Eleven-Translocases (TET 1, 2, and 3). TET enzymes demethylate DNA by producing several intermediates, 5-hydroxymethylcytosine (5hmC), 5-formylcytosine (5fC), and 5-carboxylcytosine (5CaC) [34]. In addition to being an intermediate, 5hmC in particular may have other roles in development, as studies have observed the acquisition of 5hmC by genes that are activated in neuronal cells during both postnatal developments and also during aging [35].

Mutations in TET enzymes can also contribute to the development of cancer including leukaemia [36]. While TET1 has previously been identified as a fusion protein in MLL (mixed lineage leukaemia) [37], nearly 8–23% of adult AML patients carry a mutation in TET2 [38–40]. In intermediate-risk cytogenetic AML, TET2 mutations have been associated with poor prognosis [41]. Mutations in TET2 may be an early phenomenon as they are found in approximately 20% of preleukaemic conditions such as MDS (myelodysplastic syndrome) and MPN (myeloproliferative neoplasms) [42, 43].

Direct dysfunction in enzymatic activity due to mutation in TET2 and subsequent low levels of 5hmC have been associated with myeloid tumorigenesis [44]. However, Figueroa et al. [45, 46] have suggested that it is the concomitant gain of function IDH1/2 (isocitrate dehydrogenase 1 and 2) mutations which impairs TET2 catalytic activity resulting in tumorigenesis and TET2 mutations alone do not substantially alter 5hmC levels. In addition to this both murine and human studies have shown that the loss of TET2 does not result in leukaemic transformation but does lead to preleukaemic states such as MDS through activation of downstream genes associated with self-renewal and cellular growth [47]. However, mouse models in contrast to this have shown that the loss of TET2 is sufficient for HSC self-renewal and malignant transformation, proposing that TET2 may still play a part in the leukaemogenic process [48].

### 1.5. DNMT3A Structure and Function

The DNMT3A protein is encoded on human chromosome 2p23 and is a small protein of 130 kD with several different domains [49].  Figure 2 shows the structure of DNMT3A; the human enzyme has two known splice isoforms DNMT3A1 and DNMT3A2 [50]. Expression of the two splice isoforms varies in different tissues. DNMT3A2, the shorter of the two isoforms, is predominantly expressed in embryonic stem cell found in the ovaries and testes [50], while DNMT3A1 is expressed ubiquitously in all tissues at low levels [50]. As observed with DNMT3B, transcription of DNMT3A1 occurs at two distinct promoter regions, both of which have differing levels of CpG content [51]. The control of expression of tissue specific splice forms of DNMT3A and the significance of the two promotor sites remain unclear.

As Figure 2 shows DNMT3A does not work in isolation and is seen to interact with several epigenetic modifiers through its different domains to carry out its function. Though it is unclear how DNMT3A is initially recruited to the chromatin, it is able to interact with the various types of histones at these sites [52]. The ADD (ATRX/DNMT3-DNMT3L) domain of DNMT3A may interact with histone tails to guide the enzyme to the chromatin [53]. However, interactions between histones and DNMT3A may be more complex than this. Recent data has suggested that the ADD domain may autoinhibit its own catalytic activity and through interaction with histones such as H3K4me0 (unmodified histone) this inhibition is released (Guo et al., 2015). Histones such as H3K27me3 are also observed to cover hypomethylated CpG canyons found in HSCs and are suggested to mark gene for methylation [24]. Such canyon regions may potentiate the interaction between DNMT3A and histones for functional purposes as previously discussed with respect to PRC2 gene silencing. However, as canyons are seen to be hypomethylated it is unclear how this interaction encompasses DNMT3A directly.

Several other epigenetic modifiers also bind DNMT3A for functional purposes to modify gene expression. One example of this is histone modifiers, such as H3K9 methyltransferase enzyme, which are observed to interact with DNMT3A [52], though the significance in respect to methylation activity is not well understood. Interactions between DNMT3A and SUV39H1 (histone methyltransferase) have also resulted in H3K9 methylation and subsequently reduced gene expression [54]. Another histone modifier that binds to DNMT3A through the ADD domain is EZH2 (Enhancer of Zeste homology 2) (see Figure 2). A catalytic component of PRC2, EZH2 is required for DNMT3A to bind genes that are consequently repressed by EZH2 [55]. Together these two epigenetic regulators may have a role in normal haematopoiesis, as mutations in EZH2 are seen in both MDS and myeloid malignancies [56].

Other members of DNMT family also interact with DNMT3A (see Figure 2). Methylated regions are seen to overlap for DNMT3A and DNMT3B, though it is uncertain why both enzymes are required for methylation of the same site. Some models have suggested that DNMT3A and DNMT3B work as a dimer to methylate certain satellite repeats [57]. The significance of this dimer structure is unclear; whether the dimer is required to localise satellite repeats or potentiate the DNMT3A methylating function still remains to be determined. Certain tissues which lack the accessory protein DNMT3L express both DNMT3A and DNMT3B [57], suggesting that DNMT3B may have an accessory role. When DNMT3L is coexpressed with DNMT3A, enzymatic activity is increased nearly threefold [58] but the mechanism of this is not clearly characterised. DNMTL is not seen to enhance DNMT3A binding to DNA [58] and neither does it appear to help guide DNMT3A to unmodified chromatin [53]. Further studies may identify the significance and relevance of this interaction in human tissue.

## 2. DNMT3A and Acute Myeloid Leukaemia

### 2.1. Acute Myeloid Leukaemia

AML is haematological cancer that affects the myeloid lineage and causes clonal malignant proliferation of white blood cells [68]. Inhibited differentiation and uncontrolled proliferation lead to accumulation of immature blood cells in the bone marrow; progression of this leads to cytopenia, neutropenia, and thrombocytopenia. Clinical manifestations of symptoms include fatigue, dyspnoea, susceptibility to infections, and haemorrhage. AML occurs predominantly in older adults, with more than half the cases reported in patients older than 65 years. Though rare, infrequent cases of AML in children have been reported [68]. Prognosis for AML patients is partly dependent on the age of patients; younger patients are reported to have overall better 5-year survival rate than older patients [68, 69]. Meanwhile the 5-year survival rate for those aged 65 or older is around 15–20% [68], suggesting that the mutations driving AML in older patients have greater associated lethality than those found in younger patients. Importantly this also demonstrates the devastating nature of AML as a disease.

AML is a disease of genetic heterogeneity as no single mutation is seen to drive AML yet several mutations have been identified to contribute to leukaemogenesis. Cytogenetic or chromosomal abnormalities, such as translocations, are reported in 55% of patients with AML [70]. Different cytogenetic aberrations can infer differing levels of risk and prognosis as is seen in Table 1 [59, 70]. However, cytogenetic abnormalities and molecular mutations can coincide in leukaemia patients [59]. While 40–50% of AML patients have a normal karyotype and are cytogenetically normal (CN-AML), these patients carry various molecular mutations that contribute to AML pathogenesis and as such are characterized as being intermediate-risk group [59].

**Figure 3:**
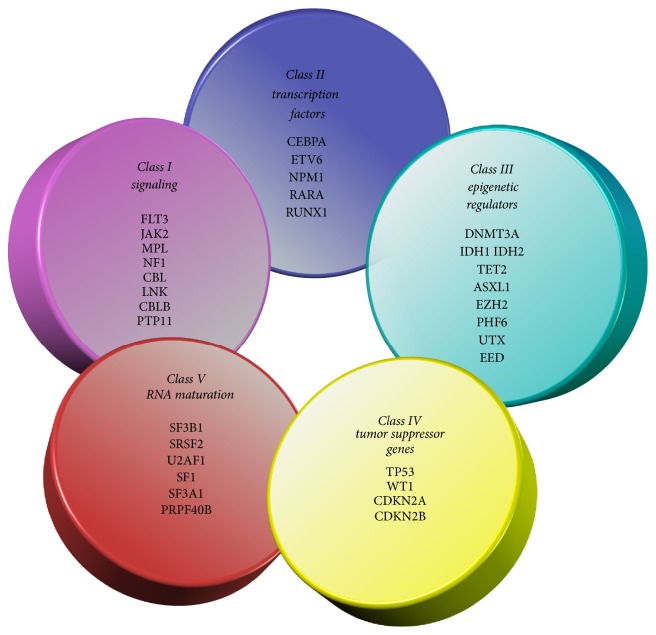
Some of the key classes of genes that are observed to contribute to the development of malignancy. All are potential targets for therapy in leukaemia. Adapted from Murati et al. [27].

The progression from single mutations in several different genes to leukaemia is a multistep process. Early simplified models for leukaemogenesis included the two-hit hypothesis, whereby two hits in two different mutation groups were needed [71]. These groups were referred to as Class I and Class II. Mutations in genes that cause uncontrolled proliferation of cells and avoidance of apoptosis were grouped as Class I mutations, while Class II mutations inhibited differentiation as seen in Figure 3.

With respect to AML this theory was supported by genomic sequencing data showing the presence of Classes I and II mutations such as FLT3 (tyrosine kinase) and NPM1 (nucleophosmin) in CN-AML patients with no other identifiable chromosomal abnormalities [72]. In addition to this, Higuchi et al. (2002) demonstrated in leukaemia mice models that a single mutation in genes did not progress to leukaemogenesis, and further mutations from other classes were required [73]. This evidence, in conjunction with the observation that no single mutation has been identified to drive AML, supported the suggestion that more than one mutation is required. Single mutations from Class II are, however, observed to cause preleukaemic conditions such as MDS [74].

However, the two-hit hypothesis is simplistic in regard to mutational groups considered. The role of mutations in epigenetic modifiers such as DNMT3A is excluded from this theory, though data is increasingly proposing that these mutations have a significant contribution to leukaemogenesis. The introduction of mutations from neither Class I nor Class II has added further complexity to understanding haematological malignancies [27]. Figure 3 shows some of these genes from different classes identified to date.

### 2.2. Epigenetic Mutations and AML

The use of more advanced whole genome and whole exome of sequencing techniques has allowed for the identification of several mutations in epigenetic regulators (see Figure 3) in AML patients. Further research into these epigenetic modifiers has enhanced our understanding of not only the function of these modifiers in normal developmental and cellular process but also the consequences of aberrant proteins in diseases such as AML. Up to 70% of de novo AML patients carry a single mutation in epigenetic modifiers such as DNMT3A and TET2 and in some cases more than one mutation in more than one epigenetic regulator [75]. Such mutations have been noted in myeloid malignancies and preleukaemic states but also in some lymphoid malignancies, notably T-cell angioimmunoblastic lymphoma [76]. Extensive research into these epigenetic modifiers has given insight into their mechanisms of action in AML and here we will focus on the current research surrounding DNMT3A mutations in AML and the theories surrounding their mechanism of action.

### 2.3. AML, DNMT3A Mutations, and DNA Methylation Patterns

Aberrant DNA methylation patterns have been observed in several studies investigating AML pathogenesis. Though global hypomethylation has been seen in various genes associated with the cellular replication, hypermethylation is also observed in specific CpG promoter regions associated with tumor repressor genes involved with differentiation and apoptosis [45, 67, 77]. In addition to this specific signature patterns of methylation have been observed across cytogenetically abnormal AML [77]. One example is observed in PML-RAR*α*-t(15;17) AML, where distinct hypomethylation and hypermethylation of gene are seen, while t(8;21) subtype AML predominant hypomethylation is observed [67]. A summary of the alterations in methylation pattern, compiled by Schoofs et al. (2014), can be seen in Table 2 [67]. It is clear from the findings shown in Table 2 that much remains unclear about the aberrant methylation patterns in relation to AML and specific mutations.

Several studies have attempted to identify the association between specific methylation patterns, prognosis, and clinical outcomes [46, 78]. Figueroa et al. [45, 46] identified that AML patients highly expressing t(8;21), inv(16), and t(15;17) had distinct methylation patterns and therefore such groups could be clustered together to predict clinical outcomes [45]. Additionally, Hájková et al. (2012) who investigated 79 AML patients and also found that high- or intermediate-risk AML patients with higher DNA methylation were seen to have better clinical outcomes [78]. In contrast to this, lower levels of methylation were associated with higher relapse and lower overall survival for AML patients [78]. Though there is some evidence that subtypes of AML have distinct aberrant methylation patterns this does, however, present the difficulty of targeting such patterns with universal epigenetic changes.

One frequently mutated epigenetic regulator in AML is DNMT3A, with approximately 22% of CN-AML patients carrying a DNMT3A mutation [79]. Though this may suggest a significant role for DNMT3A mutations in leukaemogenesis, the downstream consequence of the identified mutations in the enzyme remains largely underdetermined. While single mutations in DNMT3A do not evolve into leukaemia or alter levels of methylation, complete knockdown of DNMT3A in murine models is seen to produce cellular properties such as inhibited differentiation [80]. Challen and colleagues (2012) have suggested this was primarily due to interruptions in downstream regulators of DNMT3A such as RUNX1 (Runt-related transcription factor 1), which produce malignant properties observed with DNMT3A knockdown [80].

While it may be proposed that DNMT3A mutations contribute to altered methylation patterns in AML, how this occurs is not well characterized. Aberrant hypomethylation patterns have been noted to occur regularly in AML, specifically in non-CpG islands region, while hypermethylation of promotor CpG islands has been highly associated with mutated DNMT3A [81]. However, there is conflicting evidence regarding global methylation signatures observed in AML. Some studies have suggested no decrease in global hypomethylation [82], while other groups have identified the association of global hypomethylation patterns in respect to DNMT3A mutation, particularly affecting HOX (homeobox domain containing transcription factors) gene expression [24, 78, 81, 83]. HOX genes are a family of conserved homeodomain-containing transcription factors involved in developmental processes and are seen to have an important role in normal haematopoiesis [84, 85]. Though HOXA5, HOXA9, and PAX appear to be overexpressed due to hypomethylation in AML [81], the significance of these genes in AML remains to be determined. The overexpression of HOXA5 and HOXC4 genes, however, can ultimately affect differentiation state of myeloid cells and HSCs proliferation [81]. Further methylation studies are required to understand the impact of DNMT3A mutations on direct modulation of such genes.

While several studies have identified hypermethylation of CpG island promotor in AML [86, 87], less is known about nonpromotor sites methylation. In whole-genome studies investigating methylation patterns, predominantly hypomethylation was observed in Dnmt3a^(−/−)^ HSCs [24]. This was supported by sequential transplants of null-DNMT3A in murine models also producing global hypomethylation in progeny of null cells [80]. Specifically, loss of methylation was observed in intergenic sites in murine Dnmt3a(−/−) HSCs [88], supporting the observations in distant CpG shelves/shores and a small number of loci, in other studies [79, 81]. But what remains undetermined is the overall influence such alterations in methylation patterns have in leukaemogenesis.

### 2.4. The R882 Codon Mutation in DNMT3A

Parallel DNA sequencing techniques have identified that one of the most commonly mutated sites in DNMT3A in AML patients is the R882 hotspot [79, 83]. Approximately 60% of the mutations found in DNMT3A in AML are found to occur at this residue in the DNMT3A catalytic domain [79]. As this mutation is highly associated with poor prognosis in adults with AML [89], several studies have aimed to understand the mechanism and consequences of the R882 mutation. However, this mutation is not exclusive to just AML. Though less frequently observed, it is nevertheless present in several other myeloid malignancies and proliferative disorders such as MDS [90]. As observed with AML, MDS patients with R882 mutations are seen to have a significantly worse overall prognosis and a more rapid progression to leukaemia than those patients with non-R882 DNMT3A mutations [90]. In addition to this observation, the recent identification of other preleukaemic clones in individuals over the age of 70 has led to the theory that the R882 mutation is one that occurs early in the leukaemogenesis [91, 92].

Of the most common single codon mutation at the R882 residue is the arginine (R) to histidine (H) missense mutation [79, 83]. Holz-Schietinger and colleagues (2012) suggested that the R882H mutation formed a hypomorphic protein, whereby the DNMT3A^MUT^ produces a dominant-negative effect on the enzymatic activity of DNMT3A^WT^ [93]. Work by Kim et al. (2013) has supported this by showing that the coexpression in vitro of both DNMT3A^WT^ and DNMT3^MUT^ in heterogeneous cells produces either a gain of function action or a dominant-negative effect [94]. Around 20% of remaining function of DNMT3A^WT^ is still maintained in the presence of R882H mutations, potentially due to formation of functioning DNMT3A^WT^ dimers [93]. Disrupted dimerization by DNMT3A^MUT^ is observed to suppress half the DNMT3A^WT^ activity to produce approximately 2.5-fold reduction in methylation activity [93]. This suppression of normal activity and subsequent altered methylation could be one possible mechanism of leukaemogenesis. While the hypomethylation observed in murine models carrying the R882H mutation was initially thought to produce loss-of-function function, the successful use of the hypomethylating agent decitabine in R882 patients contraindicates this theory [95]. Rather than the R882H mutations causing increased levels of global hypomethylation, an argument could be made for the mutation alternatively causing altered patterns in methylation contributing to leukaemogenesis.

Recent data by Russler-Germain and colleagues (2014) have further demonstrated a possible in vitro mechanism of action for R882H mutation. In contradiction to previous studies, they suggested that coexpression of both DNMT3A^MUT^ and DNMT3A^WT^ is required to produce the dominant-negative effect [96]. Furthermore, rather than the dimerisation as suggested by Holz-Schietinger et al. [93], tetramers of DNMT3A^WT^ are seen to form [93, 96]. It is the loss of formation of these tetramers through the binding of DNMT3A^WT^ to DNMT3A^MUT^ in a dominant-negative fashion that is thought to be pathogenic as it produces inactive intracellular complexes [96]. Interestingly, the authors also showed that this also subsequently produced a small but significant decrease in CpG methylation [96]. While global hypomethylation of CpG islands and shores has been shown in other studies to be associated with the R882H mutation [81, 96], hypermethylation of CpG promoter regions has also been associated with the same mutation in AML [81, 83]. This gives viability to the theory that, rather than global reduction in methylation patterns, altered patterns are observed in relation to R882H mutations and the extent of severity of the mutated enzyme is ultimately dependent on the individual cellular levels of DNMT3A^MUT^ present.

Much still remains unclear regarding the R882H mutation in respect to its activity and how this mutation works in conjunction with other DNMT3A mutations that are commonly observed, as is the case with many patients who accumulate several mutations over their lifetime. Translating such findings across to AML patients is difficult, as isolating the function of single mutations in patient cells which have multitude of mutations can be challenging in terms of determining functionality. Further research would endeavour to understand the dominant-negative effect in AML patient cells. This may also provide future therapeutic solutions that would be beneficial to AML patients with this commonly mutated residue. If heterozygous patients have some level of functioning DNMT3A^WT^ present, exploiting this action through targeted inhibition of the R882 DNMT3A^MUT^ could be one avenue to pursue. However, the difficulty presented with such therapy would be the selective targeting of mutant DNMT3A over wildtype DNMT3A.

## 3. Clonal Haematopoiesis of Indeterminate Potential and DNMT3A

A crucial step in the development of premalignant and haematological malignancy is the clonal expansion of HSC carrying somatic mutations. Mutated epigenetic regulators such as DNMT3A are found in these clones of HSC and such mutations are seen to facilitate early clonal expansion in preleukemic conditions [90, 97].

**Figure 4:**
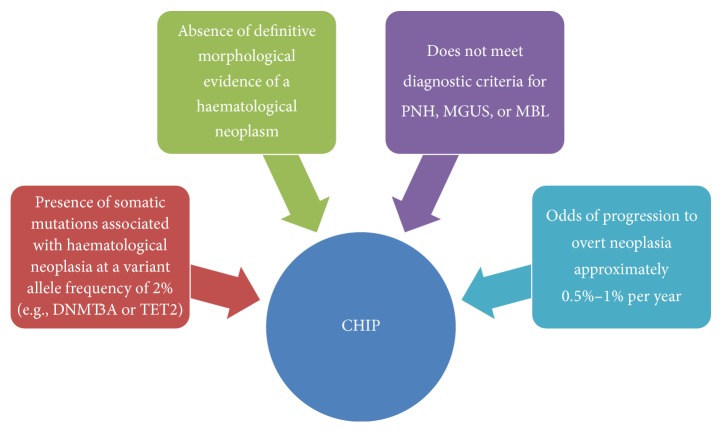
Criteria for clonal haematopoiesis of indeterminate potential. Adapted from Steensma et al. [28]. Paroxysmal nocturnal haemoglobinuria (PNH), monoclonal gammopathy of unknown significance (MGUS), and monoclonal B-cell lymphocytosis (MBL).

While mutations in epigenetic regulators have been observed in AML and preleukaemic conditions such as MDS, recent data has shown the existence of such mutations in elderly individuals in a novel preleukemic condition referred to as clonal haematopoiesis of indeterminate potential. Increasingly referred to as CHIP, this novel preleukemic condition is seen to differ diagnostically from other preleukemic conditions such as monoclonal gammopathy of unknown significance (MGUS) and monoclonal B-cell lymphocytosis (MBL) [28, 98]. The defining features of CHIP are illustrated in Figure 4. Large studies using whole-exome sequencing techniques have recently identified this phenomenon, specifically characterizing the presence of somatic mutations in three key epigenetic regulators: TET2, ASXL1, and importantly DNMT3A [91, 92]. It was concluded by both Genovese et al. (2015) and Jaiswal et al. (2014) that somatic mutations in these clones inferred an increased risk of haematological malignancy [91, 92]. Of these elderly individuals who carried mutations in DNMT3A and other epigenetic modifiers, only 4% developed lymphoid or myeloid malignancy. Though these clones carried varied DNMT3A mutations, progression to AML with DNMT3A was not observed in all cases. This is the defining criterion for CHIP, whereby the clones in CHIP carry mutations in genes notably prominent in the development of haematological neoplasms; however, the malignancy itself is absent [28].

While the mutations found in AML in DNMT3A, as previously discussed, regularly occur at the R882 region of the protein, the presence of such mutations in CHIP is undetermined. If absent or infrequent in the landscape of DNMT3A mutations identified in CHIP, it could suggest a possible theory regarding the lack of progression to myeloid malignancy. If, however, such mutations are present, this could further support the notion of R882 mutation being a founder mutation, one which is acquired in early leukaemogenesis and leads to clonal expansion.

## 4. Conclusion

The role of the epigenetic modifier DNMT3A in haematological cancers is becoming increasingly more important. The presence of key mutations in preleukemic stem cells in MDS and AML, in addition to CHIP, further supports the theory that such mutations in DNMT3A could be “founder” mutations, especially in the case of mutations found at the R882 codon hotspot. These mutations appear to arise in HSCs and play a key role in the initiation of haematological malignancy. The occurrence of such mutations has been shown to increase with age and result in clonal expansion of mutated cells. The significance of such mutations in CHIP is still unclear but the relationship between CHIP and age may suggest that such mutations are a method of adaption to the aging bone marrow environment, potentially allowing enhanced HSC self-renewal in older individuals.

What is clear from this review is that while mutations in DNMT3A play a significant role in the development of haematological disorders such as AML and MDS, understanding of the mechanisms and downstream consequences of such mutations is still undetermined. Further studies may enlighten us to the role of DNMT3A in AML and CHIP, facilitating possible therapeutic targeting in the future.

## Figures and Tables

**Table 1:** Showing the cytogenetic abnormalities that are found in AML and the current known functional consequences of these fusion proteins. Taken and adapted from Kumar et al. (2010) and individual sources as referenced below.

Translocations	Oncofusion protein	Frequency in AML	Consequence of translocation
t(8;21)	AML1-ETO	10%	Translocation involves the AML1 (RUNX1), a DNA binding TF important for haematopoietic differentiation and the ETO gene (a transcriptional repressor) to give oncofusion protein. The suggested function of the oncofusion is to exert dominant-negative effect on AML1^WT^ to suppress haematopoietic differentiation [59].
t(15;17)	PML-RAR*α*	10%	The PML-RAR*α* is expressed in haematopoietic myeloid cells and functions as a transcriptional repressor of genes involved in apoptosis, differentiation, and self-renewal [59].
inv(16)	CBF*β*-MYH11	5–8%	CBF*β*-MYH11 oncofusion protein is suggested to interact with AML1 to repress transcription in myeloid cells [59].
der(11q23)	MLL-fusions	4%	Observed in various acute leukaemia and is associated with poor prognosis. The oncofusion protein acts a potent oncogene. It directs the MLL oncoprotein targets complex to DNA sites, while fusion part works as an effector unit [59].
t(9;22)	BCR-ABL1	2%	Rare Philadelphia-positive AML [60].
t(6;9)	DEK-CAN	<1%	Chimeric fusion protein encodes a mRNA involved in leukaemogenesis [61].
t(1;22)	OTT-MAL	<1%	May regulate chromatin structure, HOX differentiation pathways, or extracellular signaling [62].
t(8;16)	MOZ-CBP	<1%	Upregulation of HOX genes and downregulation of WT1; shares similar pathway as MLL [63].
t(7;11)	NUP98-HOXA9	<1%	Inhibition of HOXA9 effecting terminal differentiation [64].
inv(3)	RPN1-EVI1	<1%	The EVI1 fusion induces gene transcription and promotes leukaemogenesis [65].
t(16;21)	FUS-ERG	<1%	Oncofusion protein that acts as a transcriptional repressor of haematopoietic specific genes [66].

**Table 2:** Effect of mutation on aberrant methylation (taken from [67]). It is clear from this table that many of the downstream consequences in relation to development of AML are still unclear. Many of these cytogenetic mutations causing AML are rare and have only been observed in a few patients to date.

Genetic alteration	Signature of DNA methylation patterns	Suggested mechanism of aberrant DNA methylation induction in AML
PML-RARa t(15;17)	Accentuated hypermethylation and hypomethylation.	PML-RARa suggested to recruit DNMTs to binding site causing DNA hypermethylation. Secondary epigenetic dysregulation as PML-RARa binds to genomic regions of epigenetic modifiers including DNMT3A.
AML1/ETO t(8;21)	Accentuated hypermethylation and hypomethylation. Though predominantly hypomethylation.	Unclear mechanism AML1/ETO may recruit DNMT1 and HDAC1. Possibly works through secondary DNA methylation disruption of AML1-ETO target genes.
CBFb-MYH11 inv(16)— t(16;16)	Predominantly hypomethylation.	Unclear mechanism.
TET2 mutations	Hypermethylation signature.	Mutated TET2 is impaired in its hydroxymethylation capacity. Unclear if DNA hypermethylated genes are direct TET2 target genes.
IDH1/2 mutations	Pronounced genome wide hypermethylation signature.	Possibly via IDH (isocitrate dehydrogenase) mutations result in DNA hypermethylation via inhibition of *α*-ketoglutarate dependent dioxygenases (e.g., TET2).
DNMT3A mutations	Genome-wide DNA hypomethylation signature: studies give mixed findings.	Mechanism of aberrant DNA methylation induction unclear. In vitro mechanism may be through loss of catalytic activity via R882H mutation. Unclear in vivo mechanism.
MLL-translocation -(11q23)	Pronounced DNA hypomethylation signature.	Unclear mechanism.
CEBP*α* mutations	Two patterns of hypomethylated and hypermethylated sites depending on the detection method used.	Unclear mechanism.
RUNX1 mutations	Discrete hypermethylation and hypomethylation signature.	Unclear mechanism.
NPM1 mutations	Mixed hypermethylation and hypomethylation pattern. Strong hypomethylation in some studies.	Unclear mechanism.
